# Mitochondrial permeability transition in redox homeostasis and ferroptosis

**DOI:** 10.1016/j.jbc.2026.113244

**Published:** 2026-06-12

**Authors:** Lishu Guo

**Affiliations:** 1 Vagelos College of Physicians and Surgeons, Columbia University, New York, New York, USA; 2 School of Medicine, Tongji University, Shanghai, China

**Keywords:** ferroptosis, mitochondria, mitochondrial permeability transition, redox homeostasis, the permeability transition pore

## Abstract

Mitochondria are major sources of intracellular reactive oxygen species (ROS), and act as central signaling hubs in maintaining homeostasis of cellular oxidative states. Mitochondrial permeability transition (MPT) is coordinately mediated by mitochondrial outer membrane permeabilization (MOMP) and opening of the permeability transition pore (PTP). MPT is highly sensitive to ROS, and serves as a critical checkpoint in redox balances and cell death. This review will summarize the regulatory systems of mitochondrial and intracellular redox homeostasis, as well as the recent advances in understanding of MPT regulatory mechanisms. Furthermore, this review highlights the functional roles of MPT in redox homeostasis and ferroptosis, a form of iron-dependent, lipid peroxidation-driven cell death. The PTP is a critical molecular switch, which can convert from a defender against mitochondrial redox stress and cell death processes, including specifically iron-dependent, lipid peroxidation-driven cell death, known as ferroptosis, into a ROS amplifier and cell death promoter depending on its open states. MOMP causes the uncoupling of the mitochondrial respiratory chain, and increases ROS production, leading to oxidative stress. The most recent work suggests that the interplay between mitochondrial carrier homolog 2 and F-ATP synthase coordinates MOMP and the PTP opening to mediate the occurrence of MPT. This review provides insights on molecular switches that regulate MPT, determining redox state and cell death.

Reactive oxygen species (ROS) play an important role in many physiological and pathological processes. When ROS are generated under normal conditions at moderate levels, they act as critical signaling molecules for modulating essential biological processes through reversible oxidative modifications of proteins (1, 2). These properly controlled ROS levels activate the adaptation behaviors of an organism under stress, which promotes cell survival, differentiation, migration, metabolism, immune responses and wound healing (3, 4, 5, 6). Excessive accumulation of ROS disrupts homeostasis of oxidative states (the redox balance) and causes irreversible damage to lipids, proteins, and DNA, leading to cellular dysfunction eventually cell death (6, 7).

Mitochondria are established as the primary cellular sites for generating ROS, specifically superoxide, which is produced during aerobic respiration. To counteract the extra ROS, mitochondria have developed adaptive antioxidant systems that are critical for maintaining redox balance (8). Mitochondria function as critical signaling hubs in regulated cell death, and orchestrate diverse death pathways. The leakage of mitochondrial intermembrane space proteins like cytochrome *c* activates caspases and results in apoptosis (9). Necrosis is a regulated form of lytic cell death triggered by different stimuli, including ATP depletion, Ca^2+^ overload and oxidative stress (10, 11). Gasdermin D permeabilization of mitochondrial membranes accelerates inflammasome-mediated pyroptosis (12). Mitochondria act as a central driver of and a defender against ferroptosis, a form of iron-dependent, lipid peroxidation-driven cell death. Mitochondria drive ferroptosis through TCA cycle activity, iron metabolism, and ROS production. In contrast, in their protective role, mitochondria guard cells against the occurrence of ferroptosis *via* antioxidant systems (13).

The mitochondrial permeability transition pore (PTP) is a high-conductance, nonselective megachannel located in the inner mitochondrial membrane (IMM) that acts as a key mediator of cell death and survival (14). The PTP is highly sensitive to the redox state, and acts as a key component in maintaining cellular redox balance (15). Its flickering opening provides a reversible pathway for the efflux of excess ROS, Ca^2+^, metabolites and other matrix molecules to the cytosol, thereby alleviating mitochondrial overload and preventing oxidative stress (16). Blunted PTP activity favors the accumulation of ROS within the matrix, inducing mitochondrial lipid peroxidation and eventually ferroptosis (17). Sustained opening of the PTP amplifies ROS production and induces mitochondrial swelling, triggering the release of cytochrome *c* from the intermembrane space (IMS) and eventually cell death (14, 16).

Mitochondrial outer membrane permeabilization (MOMP) and the PTP opening interact in a bidirectional manner, and cooperate to mediate the occurrence of mitochondrial permeability transition (MPT) (17). This review will summarize the recent advances in understanding of MPT regulatory mechanisms and highlight the roles of MPT in redox homeostasis and ferroptosis.

## Redox homeostasis and ferroptosis

### Mitochondrial redox homeostasis

Mitochondria serve as the major sites for ATP provision through oxidative phosphorylation (OXPHOS), which is critical for cell survival and metabolic homeostasis (16, 18). About 90% of intracellular ROS is generated as by-products of the respiratory chain in mitochondria of aerobic cells (6, 8, 19). Mitochondria play an essential role in cellular metabolic adaptation, biosynthesis, redox signaling by ATP generation, and regulation of ROS homeostasis (20, 21, 22, 23, 24, 25, 26).

Electrons move through the electron transport chain (ETC), generating an electrochemical H^+^ gradient that powers F-ATP synthase to produce ATP (27, 28). During electron transport, a small fraction of electrons can “leak” and prematurely react with molecular oxygen to form superoxide (29). Complexes I and III of ETC have been recognized as two major leakage points forming superoxide. Complex I releases superoxide exclusively into the matrix and is the major contributor to matrix ROS. During forward electron transfer, electrons from NADH can leak at the flavin mononucleotide site and prematurely reduce molecular oxygen to superoxide releasing into the matrix (30, 31). Reverse electron transport (RET) through Complex I acts as a substantial source of superoxide. RET can be driven by an over-reduced ubiquinone (coenzyme Q, CoQ) pool and high proton motive force, which forces electrons to flow backward from CoQH_2_ to Complex I, generating bursts of ROS at the CoQ-binding site located on the matrix side (30, 31). Complex III can release superoxide into both the IMS and matrix (32).

**Figure 1:**
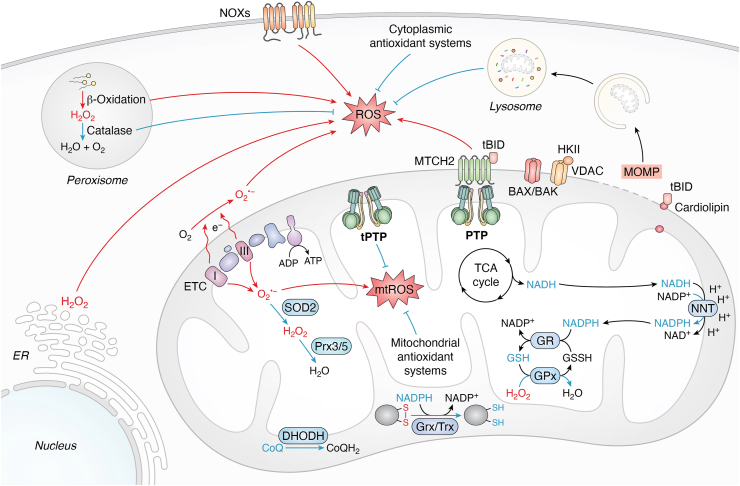
**Mitochondria are major sources of intracellular ROS and act as a redox sink.** Mitochondria are established as the primary cellular sites for generating ROS. During electron transport through electron transport chain, a small fraction of electrons leaks and react with O_2_ to form superoxide. Reverse electron transport through Complex I acts as a greater source of superoxide. NADPH oxidases are membrane-bound sources that generate ROS at the cellular surface. Peroxisomes generate hydrogen peroxide as byproducts of breaking down fatty acids specifically through β-oxidation. Endoplasmic reticulum generates significant amounts of peroxide during oxidative protein folding. The nuclear environment maintains its own specific redox homeostasis to protect genetic material and regulate gene expression. MOMP is tightly regulated by members of the BCL-2 family such as BAX/BAK, tBID, BCL-2, and other mitochondrial components including MTCH2, VDAC, hexokinases, particularly hexokinase II, cardiolipin. MOMP causes the uncoupling of the mitochondrial respiratory chain, promoting ROS production and oxidative stress. Peroxisomes contain high concentrations of the enzyme catalase, which immediately converts hydrogen peroxide into H_2_O and O_2_. Lysosomes degrade damaged mitochondria through mitophagy, and oxidized components through autophagy, thereby reducing the intracellular burden of oxidative stress. Mitochondria develop adaptive antioxidant systems to counteract the extra ROS. Mitochondrial antioxidant enzymes include superoxide dismutase, Prx3/5, glutathione peroxidase, glutaredoxin, thioredoxin, nicotinamide nucleotide transhydrogenase, *etc.* Mitochondrial low molecular weight antioxidants include GSH, CoQ and NAD(P)H. Mitochondrial enzymes that prevent mitochondrial lipid peroxidation include glutathione peroxidase and dihydroorotate dehydrogenase. Mitochondrial NADH is generated in the TCA cycle, nicotinamide nucleotide transhydrogenase relies on proton gradient to regenerate NADPH from NADP^+^ utilizing NADH. The sustained opening of PTP induces mitochondrial dysfunction, amplifies ROS production and depletes antioxidant systems, leading to severe oxidative stress. While the tPTP minimizes ROS production and prevents excessive ROS accumulation in the matrix, functioning as a mitochondrial defense system against oxidative stress. ROS, reactive oxygen species; MOMP, mitochondrial outer membrane permeabilization; tBID, truncated BID; VDAC, voltage-dependent anion channel; MTCH2, mitochondrial carrier homolog 2; HKII, hexokinase II; ; Prx, peroxiredoxin; GR, glutathione reductase; TCA, tricarboxylic acid; PTP, the mitochondrial permeability transition pore; tPTP, transient or flickering opening of the PTP; BH, BCL-2 homology.

Mitochondria develop adaptive antioxidant systems to counteract the extra ROS, which are essential for maintaining the critical redox balance (Fig. 1). The matrix superoxide can be rapidly converted by mitochondrial superoxide dismutase into hydrogen peroxide (H_2_O_2_), a more stable ROS (33). Peroxiredoxins often act as the primary scavenger for low levels of H_2_O_2_, generating moderate H_2_O_2_ signaling rather than toxic levels (34). Prx3 and Prx5 are located in the matrix, while Prx3 handles up to 90% of mitochondrial H_2_O_2_ (34, 35). GSH peroxidase (GPx) functions as a critical antioxidant system in both the matrix and the IMS (36). H_2_O_2_ is able to be converted into non-toxic H_2_O by GPx, while GSH is converted into oxidized GSSG (37). Mitochondrial glutathione reductase reduces GSSG back to GSH using NADPH as an electron donor for maintaining the antioxidant capacity within mitochondria (36). Oxidized thiols can be reduced by enzymes of the glutaredoxin (Grx) and thioredoxin (Trx) systems in the IMS (38). Mitochondrial GSH peroxidases including GPx4 and GPx1 are essential for scavenging H_2_O_2_ and detoxifying lipid hydroperoxides directly in mitochondrial membranes (39, 40). Dihydroorotate dehydrogenase located in the IMM reduces CoQ to CoQH_2_ and acts independently of the mitochondrial GPx4 (mGPx4) to prevent mitochondrial lipid peroxidation (41).

NADPH is essential for maintaining the activity of antioxidants in the matrix, acting as the primary electron donor to drive antioxidant systems like GSH and Trx reductases to neutralize ROS (42). Therefore, the mitochondrial matrix maintains a high NADPH/NADP^+^ ratio to fuel the antioxidants that detoxify ROS like H_2_O_2_, which is central to metabolism and redox signaling (43). Nicotinamide nucleotide transhydrogenase (NNT) serves as a primary source of mitochondrial NADPH, and acts as a key link transferring electrons from NADH to NADPH. NNT relies on proton gradient across the IMM to regenerate NADPH from NADP^+^ utilizing NADH (43, 44). Mitochondrial NADH can be generated in the TCA cycle and imported from the cytoplasm through the malate–aspartate shuttle (45, 46). NADH is oxidized by Complex I, which is coupled to proton pumping from the matrix into the IMS, establishing the proton motive force and driving F-ATP synthase to produce ATP (47).

In addition to NNT, isocitrate dehydrogenase 2, glutamate dehydrogenase and malic enzyme 3 are NADP^+^-dependent enzymes that also contribute to replenishing mitochondrial NADPH (48). Mitochondrial serine catabolism is another important source of NADPH through one-carbon metabolism (49, 50). Methylenetetrahydrofolate dehydrogenase 2 use 5,10-CH2-THF and NADP^+^ as substrates to produce 5,10-methenyl-tetrahydrofolate (5,10-CH = THF) and NADPH (49, 50). The mitochondrial folate pathway is linked to the cytoplasm through the transport of one-carbon units, allowing mitochondria to transport electrons across the IMM essentially facilitating the conversion of NADP^+^ to NADPH in the matrix (51).

### Intracellular redox homeostasis

Mitochondria and NADPH oxidases (NOXs) are established as the primary sources of ROS in cell signaling, playing critical, synergistic roles in maintaining cellular homeostasis (52). Mitochondrial ROS (mtROS) are generated as byproducts of metabolism, while NOXs produce ROS directly at the membrane in response to specific stimuli (52). NOXs are membrane-bound sources involved in the localized generation of ROS at the cellular surface (53, 54). Mitochondria-derived superoxide can activate NOXs, triggering a localized and sustained burst of ROS (55). This cross talk between mitochondria and NOXs represents a feed-forward vicious cycle of ROS production (54, 55). Mitochondria possess the highest levels of antioxidants in the cell, which break this vicious cycle, reducing both mtROS production and NOX activity (54).

Mitochondria and peroxisomes play a key role in both the production and scavenging of ROS, acting as central hubs for redox signaling and metabolic homeostasis (56, 57). Peroxisomes generate H_2_O_2_ as byproducts of fatty acid breakdown, particularly though the process of β-oxidation (58). Peroxisomes contain high concentrations of the enzyme catalase, which immediately converts H_2_O_2_ into water and oxygen (59). The peroxisome counteracts oxidative stresses by suppressing catalase import *via* Pex14 at Ser232 induced by H_2_O_2_, thereby enhancing cellular resistance to oxidative distress by the increase of cytosolic catalase (60). Mitochondria and peroxisomes are metabolically linked organelles that share key fission machinery including FIS1, MFF, and DRP1 (61). Peroxisomes perform initial oxidation of very-long-chain fatty acids and polyunsaturated fatty acids, and the resulting products are further metabolized by mitochondria (62). Peroxisomes form contact sites with mitochondria to facilitate the transfer of ROS, and directly mitigate mtROS (63). Increased abundance of peroxisomes assists in supporting this mitochondrial metabolic shift, further reducing ROS levels and promoting cell survival (64).

Endoplasmic reticulum (ER) is another, often overlooked, source of cellular ROS, generating significant amounts of peroxide during oxidative protein folding (65, 66). The ER is a crucial organelle for protein folding, lipid synthesis, and calcium storage, which is highly sensitive to changes in intracellular homeostasis (65, 67). When the protein folding capacity is overwhelmed due to mutation, nutrient deprivation, or environmental toxins, misfolded proteins accumulate, triggering ER stress and the unfolded protein response (68, 69). ROS are significant byproducts of unfolded protein response -regulated oxidative folding machinery in the ER (65). Endoplasmic oxidoreductin-1 oxidizes protein disulfide isomerase to regenerate its active oxidized form, which then facilitates disulfide bond formation in nascent proteins (65, 70, 71). ROS are generated in the ER lumen largely due to Endoplasmic oxidoreductin-1 mediated electron transfer from protein disulfide isomerase to molecular oxygen, particularly during oxidative protein folding (70, 71). Increased mitochondrial respiration and biogenesis serve as a critical adaptive, pro-survival mechanism during ER stress by reducing ROS (64, 72).

The nucleus is only a minor site of ROS generation, but it functions as a distinct, tightly controlled redox-sensitive compartment (2, 73). The nucleus regulates gene expression, DNA repair, and protein function through redox-dependent mechanisms including cysteine oxidation and nuclear import/export modulation (73, 74). To counteract ROS-induced severe DNA damage, the nucleus maintains a highly reduced environment by antioxidant systems, notably GSH and Trx (75, 76). In response to oxidative stress, SOD1 relocates to the nucleus, acting as both an antioxidant enzyme and a transcription factor to maintain low ROS levels and ensure genomic stability (77). Elevated ROS oxidizes redox-sensitive cysteine residues on Kelch-like ECH-associated protein 1, resulting in dissociation of nuclear factor erythroid 2-related factor 2 from its inhibitor Kelch-like ECH-associated protein 1. Nuclear factor erythroid 2-related factor 2 then translocates to the nucleus and binds to the antioxidant response element, and initiates transcription of cytoprotective and detoxifying enzymes (75). Mitochondria relocate to the perinuclear region during cellular stress, which allows mtROS to reach the nucleus and oxidize transcription factors, promoting stress-adaptive responses (78, 79).

Lysosomes maintain intracellular redox homeostasis by functioning as central signaling hubs and degradation centers (80). Autophagy may also protect against oxidative stress by facilitating the turnover of oxidized components including proteins and lipids (81). Lysosomes degrade damaged, ROS-producing mitochondria through a specialized form of autophagy called mitophagy, thereby reducing the intracellular burden of oxidative stress (82). Lysosomes function as redox-sensitive signaling hubs by integrating nutrient availability and metabolic stress, acting as a platform for the mTORC1 complex to regulate cell growth and autophagy (83).

Mitochondria are major sources of intracellular ROS and also function as a dynamic redox buffer (redox sink) (Fig. 1). Mitochondria are foundational to intracellular redox homeostasis, and they are central hubs that balance ROS generation, antioxidant defenses, and metabolic signaling in response to environmental changes (3, 84, 85). Mitochondria constantly communicate with other organelles through direct physical contacts and functional signals, such as metabolite exchange, ROS, and Ca^2+^ signaling, allowing them to coordinate essential processes (18, 86).

### Ferroptosis

Ferroptosis is a form of regulated cell death characterized by iron-dependent lipid peroxidation, morphologically and biochemically distinct from apoptosis, necrosis, and autophagy (87, 88, 89). Unlike classical programmed cell death pathways, ferroptosis is primarily driven by the accumulation of lethal lipid ROS and impaired antioxidant defenses, resulting in excessive iron-catalyzed oxidation of membrane phospholipids and distinct morphological features (88, 89, 90, 91). Excess intracellular iron catalyzes the Fenton reaction, producing ROS that induce severe peroxidation of polyunsaturated fatty acids in membranes (88, 89, 90, 91). Ferroptotic cells exhibit distinctive ultrastructural changes, most notably mitochondrial shrinkage with reduced mitochondrial volume, increased membrane density, destruction of the cristae, and rupture of the outer mitochondrial membrane (OMM) (87, 88, 89). Ferroptosis functions as a critical nexus between iron dysregulation, lipid metabolism, and redox imbalance (88, 89, 90, 91).

GPx4 is a selenoenzyme that contains a selenocysteine residue in its catalytic center. GPx4 uses GSH as a co-factor to neutralize lipid hydroperoxides within cell membranes under oxidative stress (92, 93, 94). GPx4 is considered to be the most critical enzyme for preventing lipid hydroperoxide accumulation and inhibiting ferroptosis (88, 95). GPx4 exists as three distinct isoforms: mitochondrial (mGPx4), nucleolar/nuclear (nGPx4) and cytosolic (cGPx4) (39). mGPx4 specifically safeguards mitochondrial phospholipids and integrity, such as cardiolipin and other polyunsaturated fatty acid-containing lipids, which are highly susceptible to oxidative damage induced by mtROS (13). Upon GSH depletion, direct inhibition, or oxidative stress, mGPx4 activity is impaired and thus lipid peroxides accumulate, leading to mitochondrial dysfunction, increased ROS production, and amplification of ferroptotic signaling (13).

Several GPx4-independent and parallel regulatory pathways for ferroptosis have been identified (96). Ferroptosis suppressor protein 1, also known as apoptosis inducing factor mitochondria associated 2, reduces ubiquinone using NADPH to suppress ferroptosis (97, 98). Dihydroorotate dehydrogenase is a flavin-dependent mitochondrial enzyme that protects mitochondrial membranes from ferroptotic lipid damage by supporting ubiquinone reduction within the IMM (41). SLC25A39 is essential for mitochondrial GSH import and its protein abundance is negatively regulated by GSH availability, thus potentially mediating ferroptosis (99). The deletion of SLC7A11, a vital component of the cystine/glutamate reverse transporter, induces pancreatic tumor ferroptosis in mice (100). P53 can inhibit cystine uptake by inhibiting the expression of SLC7A11 thereby enhancing the sensitivity of cells to ferroptosis (101). Therefore, cellular defense against ferroptosis is a collaborative, multilayered, and context-dependent system across different cellular compartments, allowing cells to resist ferroptotic stress even when GPx4 activity is compromised (96).

## Mitochondrial permeability transition

### Mitochondrial outer membrane permeabilization

Under physiological conditions, the OMM is permeable to small metabolites and ions through channels such as voltage-dependent anion channel (VDAC), allowing the exchange between the cytosol and the IMS, which is required for cellular respiration (102). In contrast, when the OMM becomes highly permeable, MOMP is a central event in the intrinsic pathway of apoptosis, acting as the point of no return that commits a cell to programmed death (103, 104). During apoptotic signaling, the OMM becomes permeable, allowing the release of pro-apoptotic proteins, such as cytochrome *c*, from the intermembrane space into the cytosol (103, 104). Therefore, the OMM provides an interface with the rest of the cell, and its permeability is strictly controlled to maintain mitochondrial health or induce cell death when necessary (102, 105). Oxidative stress, characterized by elevated ROS, can act as a signal for initiating MOMP, which promotes further mitochondrial damage and causes a surge in mtROS (15, 75).

MOMP is tightly regulated by members of the BCL-2 family, which include pro-apoptotic proteins, such as BAX and BAK, and anti-apoptotic proteins, such as BCL-2 and BCL-XL (106, 107). The BCL-2 family is defined by four conserved BCL-2 homology (BH) domains, named BH1, BH2, BH3, and BH4, which determine their pro-survival or pro-apoptotic functions in the regulation of cell death (107). The BCL-2 family proteins can be divided into three subgroups based on the function and BH domain composition: multi-domain anti-apoptotic proteins contain BH1-4, multi-domain pro-apoptotic proteins lack BH4 but retain BH1-3, and BH3-only pro-apoptotic proteins possess solely the BH3 domain (106).

Even though BH1 and BH2 domains are primarily found in anti-apoptotic proteins to promote survival, they are also present in some pro-apoptotic proteins, where they may assist in protein-protein interactions that allow homodimerization or heterodimerization (108, 109). The BH3 domain is a crucial death domain that acts as a binding motif, allowing BH3-only proteins, such as BID, Bcl-2-interacting mediator of cell death (BIM), BAD, to activate pro-apoptotic proteins BAX/BAK or inhibit anti-apoptotic proteins like BCL-2 (110). The BH4 domain of BCL-XL is crucial for suppressing apoptosis and distinguishing anti-apoptotic BCL-2 members from pro-apoptotic ones, as its deletion or inhibition converts them into pro-apoptotic molecules (111, 112, 113). The anti-apoptotic members possess BH1-4, while the pro-apoptotic proteins are either effector including BAX, BAK with BH1-3 or BH3-only proteins (114, 115, 116).

BH3-only activator proteins, including BIM, truncated BID (tBID) and p53 upregulated modulator of apoptosis, have traditionally been considered to be essential and direct initiators of apoptosis (117, 118). BH3-only activators have been proposed to directly bind to BAX or BAK, prompting a conformational change that allows them to form symmetric homodimers that further oligomerize and form pores in the OMM, triggering MOMP (119, 120). Although BH3-only proteins can promote the activation of BAX/BAK, accumulating evidence suggests that they are not necessary for BAX/BAK activation (121, 122). Recent studies demonstrate that BH3-only activators can bind to and neutralize anti-apoptotic proteins, which would otherwise indirectly promote BAX/BAK activation (106, 123). BH3-only sensitizers, such as BAD, NOXA, and HRK bind to specific anti-apoptotic proteins, preventing them from sequestering direct activators or activated BAX/BAK (114, 124).

Beyond the interplay between BCL-2 family proteins, mitochondrial components, such as VDAC, metabolic enzymes especially hexokinases, membrane lipids notably cardiolipin and mitochondrial carrier homolog 2 (MTCH2), act as crucial regulatory platforms for assembling, sequestering, or activating BAX/BAK in response to apoptotic stimuli (Fig. 1) (125, 126, 127, 128, 129, 130, 131). These components together with BCL-2 family help define the apoptotic threshold of a cell and control MPT, governing how quickly and strongly a cell responds to death signals (125, 126, 127, 128, 129, 130, 131, 132).

**Figure 2:**
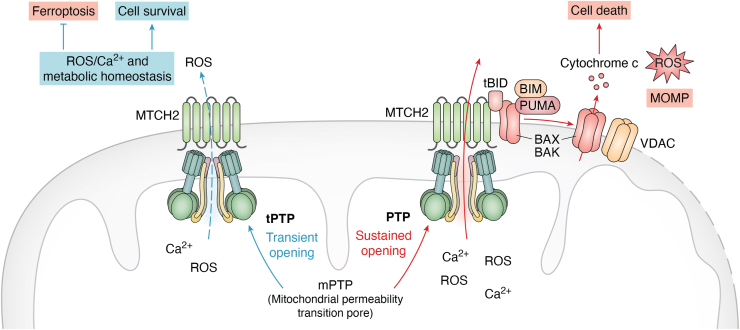
**The PTP is a critical molecular switch, which can convert from a defender against mitochondrial redox stress and ferroptosis into a ROS amplifier and cell death promoter depending on its open states.** The BCL-2 homology 3-only proteins, including BID (tBID), Bcl-2-interacting mediator of cell death, and p53 upregulated modulator of apoptosis, promote activation of the pro-apoptotic effectors BAX and BAK, thereby driving MOMP. MTCH2 acts as a crucial OMM receptor that recruits tBID to the mitochondria. The binding of tBID induces BAX to oligomerize and form pores in OMM. VDAC1 can also oligomerize and form large pores, triggering MOMP. VDAC2 is required for efficient BAX-mediated MOMP and apoptosis. The sustained opening of PTP causes matrix swelling and rupture of OMM, leading to subsequent MOMP. MOMP allows the release of proapoptotic factors like cytochrome *c* to enter the cytosol, leading to cell death. MTCH2 interacts with F-ATP synthase and promotes the enzyme dimerization, thus regulating the PTP activity. The tPTP allows the release of ROS from mitochondria into cytosol, preventing mitochondrial lipid peroxidation. The tPTP contributes to ROS/Ca^2+^ homeostasis, and acts a mitochondrial defense system against ferroptosis. PTP, the mitochondrial permeability transition pore; tPTP, transient or flickering opening of the PTP; ROS, reactive oxygen species; MTCH2, mitochondrial carrier homolog 2; OMM, outer mitochondrial membrane; tBID, truncated BID; BH, BCL-2 homology; VDAC, voltage-dependent anion channel; MOMP, mitochondrial outer membrane permeabilization; PTP, permeability transition pore.

VDAC possesses three isoforms in mammals, which controls metabolic flux and acts as a docking site for cell death regulators (102). VDAC1 is the most abundant isoform and handles general metabolic flux, thereby playing a crucial role in maintaining cellular metabolism and regulating apoptosis (133). VDAC1 can act as a target for the members of the BCL-2 family. Interaction of VDAC1 with the pro-apoptotic proteins BAX/BAK and BIM leads to MOMP. On the other hand, interaction of VDAC1 with the anti-apoptotic proteins BCL-2 and BCL-XL inhibits VDAC1 oligomerization, the assembly process that proteins cluster together and form multi-subunit structures, thereby preventing MOMP (133, 134, 135). Under oxidative stress conditions, VDAC1 can oligomerize and form large pores, triggering MOMP, that allow the release of mtDNA fragments (136). VDAC2 interacts with the inactive conformer of BAK, and inhibits BAK activation (137). BH3-only proteins displace VDAC2 from BAK, enabling homo-oligomerization of BAK and apoptosis (137). VDAC2 is required for efficient BAX-mediated apoptosis but not for BAK, revealing a functional difference between BAX and BAK impacted by their interactions with VDAC2 (Fig. 2) (129, 137). VDAC3 is crucial for maintaining metabolic pathways, calcium homeostasis, redox balance, and sperm function (138). VDAC3 protects mitochondria from oxidative stress by acting as a redox sensor, specifically through its unique cysteine residues (139).

Hexokinases, particularly hexokinase II (HKII), regulate MOMP by interacting with mitochondria and modulating apoptotic signaling (126). VDAC acts as the primary anchoring site on the OMM for HKII (140). HKII catalyzes the first step of glycolysis, and it binds to VDAC, positioning it to preferentially utilize ATP generated by mitochondria for phosphorylating glucose (141). HKII competes with pro-apoptotic proteins, such as BAX and BAK, for binding sites on VDAC, and thus HKII acts as an anti-apoptotic factor when it binds to VDAC (140). By occupying VDAC, HKII prevents the oligomerization of pro-apoptotic proteins BAX and BAK and MOMP (126). Hexokinases can also facilitate the retrotranslocation of pro-apoptotic proteins BAX, BAK, and tBID from the mitochondria into the cytosol (126). Under cellular stresses including oxidative stress, metabolic disruption, or inhibition of survival signaling pathways like the AKT pathway, HKII detaches from VDAC, facilitating BAX/BAK activation and pore formation (126, 133). The association of HKII with the OMM acts as a critical checkpoint, directly linking enhanced aerobic glycolysis to cell survival (142).

Cardiolipin is a key anionic phospholipid uniquely located and synthesized in the IMM (143). It is essential for mitochondrial structure and membrane stability, and optimizes the function of respiratory chain complexes (144, 145). Cardiolipin is rich in unsaturated fatty acids, making it highly susceptible to ROS-induced oxidation, which causes mitochondrial dysfunction and initiates apoptosis (146). Under stress, cardiolipin redistribution to the OMM acts as a binding site for tBID, promoting tBID-mediated BAX activation and membrane permeabilization (147). During apoptosis, Bax translocates from the cytosol to the OMM, while cardiolipin plays a key role in facilitating the recruitment of Bax and its perforation (148). Cardiolipin-deficient cells exhibit impaired apoptosis in response to Fas stimulation, because cardiolipin acts as an essential anchoring and activating platform for caspase-8 on the OMM (149). Cytochrome *c* acts as a cardiolipin oxygenase, and oxidized cardiolipin is required for the release of proapoptotic factors (150). The dispatch of the intrinsic apoptotic pathway is accompanied by early mitochondrial production of mtROS, which is an important feature of the apoptotic program (150).

MTCH2, also known as Met-induced mitochondrial protein, acts as a crucial outer membrane receptor that recruits tBID to the mitochondria, enabling MOMP and apoptosis (151). It has been shown that MTCH2 can compensate for the lack of cardiolipin, as cardiolipin and MTCH2 can have redundant roles in recruiting tBID (152). MTCH2 specifically interacts with tBID, facilitating its activity in the intrinsic apoptosis pathway and death-receptor signaling (151, 152, 153, 154, 155). Fluorescence anisotropy studies identified that tBID binds to MTCH2 *via* two main regions: tBID residues 59 to 73 interact with MTCH2 residues 140 to 161, and tBID residues 111 to 125 interact with MTCH2 residues 240 to 290 (156). The binding of MTCH2 to tBID at the OMM promotes tBID translocation to mitochondria from the cytosol. Once bound to MTCH2, tBID undergoes slow structural rearrangements to a primed state, which is a prerequisite for interaction with BAX (155, 157). The interaction of tBID with BAX induces its conformational activation, followed by insertion and oligomerization in the OMM, thereby driving MOMP (157). MTCH2’s role extends to promoting the interaction between tBID and BAX, and the tBID-BAX interaction triggers MOMP (Fig. 2) (151, 152, 153, 154, 155, 156, 157). Mitochondrial-localized tBID, whose association with mitochondria is facilitated by MTCH2, can also promote MOMP through neutralization of anti-apoptotic BCL-2 family proteins and facilitation of BAX activation (121, 151).

### Mitochondrial permeability transition pore

The PTP is a high-conductance, non-selective channel in the IMM, and its opening allows the uncontrolled diffusion of matrix molecules, and may eventually causes mitochondrial depolarization, swelling, and apoptosis (14, 16, 158). Transient or flickering opening of the PTP (tPTP) acts as a rapid efflux valve; it therefore functions as a protective mechanism to maintain cellular ROS and Ca^2+^ homeostasis (158, 159, 160). In contrast, sustained PTP opening leads to mitochondrial swelling rupture of the OMM, and eventually cell death (158, 159, 160).

The open-closed transitions of PTP are highly regulated by multiple effectors. The opening of PTP can be activated by cyclophilin D (CyPD), matrix Ca^2+^, ROS, NAD(P)^+^, inorganic phosphate (Pi), alkaline pH, and low mitochondrial transmembrane potential (Δψm) (161, 162, 163, 164, 165, 166, 167, 168, 169, 170, 171, 172, 173, 174, 175, 176, 177). While its opening can be inhibited by cyclosporin A (CsA), matrix Mg^2+^/Sr^2+^/Mn^2+^/Ba^2+^, GSH, NAD(P)H, ADP, acidic pH, high Δψm (161, 162, 163, 164, 165, 166, 167, 168, 169, 170, 171, 172, 173, 174, 175, 176, 177). Mitochondrial matrix Ca^2+^ is essential for the PTP formation, and its binding promotes the pore opening, while its matrix binding site is occupied by other divalent cations such as Mg^2+^, Sr^2+^, Mn^2+^, Ba^2+^ leading to an inhibitory effect on the PTP (158, 162).

Binding of CyPD to PTP sensitizes the pore to Ca^2+^, while its detachment following binding to CsA suppresses pore opening (178, 179, 180). Pi favors the interaction of CyPD with the pore, thus acting as an inducer of the mammalian PTP (167, 168). Acidic matrix pH inhibits the PTP through protonation of histidyl residues (172). The PTP is a voltage-dependent channel, with high Δψ_m_ favoring the closed conformation and depolarization leading to the open conformation (170). The oxidation of critical thiol (SH) groups on matrix side promotes PTP-dependent Ca^2+^ efflux, which can be prevented by their reaction with N-Ethylmaleimide (NEM) (181, 182). The oxidation of matrix pyridine nucleotides, specifically NADPH and NADH, leads to a more oxidized environment, which alters the SH groups to form disulfide (S-S) bonds, inducing an open conformation of the PTP (183). The depletion of GSH promotes the PTP opening induced by H_2_O_2_ (174). PTP opening is also favored by oxidation of GSH to GSSG, indicating that the pore is regulated by the redox state of the mitochondrial GSH pool and is an established target of oxidative stress (175).

The mitochondrial ATP synthase, also termed F-ATP synthase or complex V, is a rotary molecular motor responsible for the synthesis of ATP driven by the proton electrochemical gradient (184). Mammalian F-ATP synthase is a multisubunit complex composed of F_1_ and F_O_ motors, which are linked by central and peripheral stalks (14, 184). The rotation of the membrane-embedded F_O_ sector causes conformational changes in the catalytic F_1_ sector to produce ATP that releases into the matrix (14, 184). A soluble F_1_ head domain consists of subunits α and β with a 3:3 stoichiometry (fixed ratio), which are organized into a α_3_β_3_ hexamer (185). The rotary central stalk subunit γ interacts with subunits δ and ε at the foot that serves as the base and stabilize the interaction between the stalk and the c-ring (186). The peripheral stalk acts as a stator that links F_1_ sector to F_O_ domain. The stator is composed of subunits b, d, coupling factor 6 (F_6_) and oligomycin sensitivity conferral protein (OSCP) (187). N-terminus of OSCP binds to the uppermost region of the stator *via* the N-termini of the three subunits α, while the C-terminus of OSCP interacts with the C-terminus of subunit b (188). The α-helix of subunit b, stiffened by subunits F_6_ and d, protrudes to the IMM (188). The F_O_ domain consists of subunits c-ring, ATP6 (or subunit a), ATP8 (or A6L), diabetes-associated protein in insulin-sensitive tissue, 6.8 kDa proteolipid (or 6.8 PL or ATP5MPL or ATP5MJ or subunit j), f, e and g (189). Subunits e, f, g, j and diabetes-associated protein in insulin-sensitive tissue contribute to the stability of F-ATP synthase dimers (190). Subunits f, g and e interact with subunit b, and form a compact triple transmembrane helix bundle, which is stabilized by several highly conserved salt bridges including eR15-gE82, gE72-bK28 and gE91-bK48 (191). This group of subunits acts like a “hook apparatus”, providing the link from the lateral stalk to the extended C terminus of subunit e, which is anchored to a lipid plug capping the c-ring (191). In the dimeric complex, the two j subunits interact with each other across the monomer-monomer interface (192). N-terminus of subunit j and N-terminal loop of subunit f form the closest contact sites at the matrix side. The C terminus of subunit e is anchored to a lipid plug capping the c-ring and interacts with the C-terminal ends of the 6.8 PL helices at the IMS side (191, 193).

F-ATP synthase inhibitory factor 1 (IF1) is a natural inhibitory protein of F-ATP synthase (194), and its binding to F-ATP synthase is determined by mitochondrial matrix pH (195). The dimeric IF1 bridges the F1-F1 sector and promotes the dimerization of F-ATP synthase (196). The dimeric complexes can further form tetramers, which are stabilized by subunits e, g, j and IF1 (18).

CyPD is the mitochondrial receptor for CsA and a well-characterized regulator of the PTP; it thus acts as an important cue to explore the regulatory mechanism of PTP (17). CyPD ablation causes a higher threshold for Ca^2+^- and oxidative stress-induced PTP opening, but the PTP can still open with higher Ca^2+^ loads, indicating that CyPD is a critical activator of the PTP but not a structural component of the pore itself (197). The OSCP subunit of F-ATP synthase has been identified as a binding site for CyPD (167, 168). Purified F-ATP synthase forms a Ca^2+^-dependent and voltage-sensitive high-conductance channel with properties matching the PTP (168, 198, 199, 200, 201). KO of yeast subunit e or g inhibits dimerization of F-ATP synthase, desensitizes the PTP to Ca^2+^ and prevents high-conductance channel formation (199, 202). Knockdown of subunit f destabilizes F-ATP synthase dimers, decreasing the size of PTP and its sensitivity to Ca^2+^ (203). Knockdown of subunit j doesn’t interrupt F-ATP synthase dimer formation, but strikingly increases the Ca^2+^ loads required to open the pore (17). Overexpression of IF1 promotes MPT *via* opening of the PTP, while ablation of IF1 desensitizes the pore to Ca^2+^ and prevents cell death induced by oxidative stress (204). IF1 interacts with p53-CyPD complex that may causes a conformational change transmitted to PTP *via* OSCP, favoring the pore formation (204). T163S mutation of the subunit β desensitizes the PTP to Ca^2+^ and protects from PTP-dependent cell death (205). Ca^2+^ binding to the catalytic core causes a conformational change that is propagated to the inner membrane by the peripheral stalk *via* OSCP, and thus matrix Ca^2+^ is essential for PTP formation (158). H112Q and H112Y mutations of subunit OSCP abolish the sensitivity of PTP to matrix acidification in human HEK293 cells (206). C141S mutation of subunit OSCP protects against the toxicity of oxidant diamide in the absence of CyPD (207). The inhibitory or inducing effects on the PTP by the modification with arginine-specific glyoxals depend on the net electric charge and hydrogen bonding of the resulting arginine adducts; thus, the target arginine is supposed to play a role as a voltage sensor (208). Arg-107 of yeast F-ATP synthase subunit g mediates sensitivity of the PTP to phenylglyoxal (209). Arg-8 of subunit e interacts with Glu-83 of subunit g favoring the stability of F-ATP synthase dimers and generation of the full-conductance PTP (200). PTP may be originated from a Ca^2+^-dependent conformational change within the catalytic core transmitted to subunits g/e through lateral stalk *via* OSCP, allowing subunit e to pull the lipid plug out of the c-ring (17, 191, 210).

The adenine nucleotide translocase (ANT) acts as a transporter that exchanges ADP for ATP across the IMM (211). Ca^2+^-induced MPT can be activated and inhibited by the ANT inhibitors atractyloside and bongkrekic acid, respectively, leading to the hypothesis that ANT is a pore-forming component of PTP (212). ANT interacts with CyPD and forms a CsA-sensitive channel, which acts as a distinct permeability pathway (213, 214). In the triple ANT KO cells, the sensitivity to CsA is still present and MPT can still occur but more Ca^2+^ is required (215, 216). ANT acts as a distinct CsA-sensitive low-conductance channel and F-ATP synthase contributes to the formation of high-conductance megachannels (210, 213, 214).

### Cooperative functions of MOMP and the PTP opening

MOMP and the PTP opening interact in a bidirectional manner, and function as two distinct but interrelated mechanisms of MPT. MOMP can occur without significant matrix swelling, characterized by the release of cytochrome *c* while the IMM remains intact (217). However, MOMP disrupts mitochondrial structure and destabilizes the membrane environment, resulting in the uncoupling of OXPHOS and a subsequent depletion of ATP (218). MOMP often disrupts electron transport and depletes Δψm, leading to increased mtROS production and oxidative stress, which further causes ROS-induced ROS release (RIRR) (15, 218). The OMM acts as a critical regulatory barrier that controls the access of stress signals, such as Ca^2+^, ROS, and metabolites, to the IMM. VDAC is central to mediate Ca^2+^ uptake and to regulate the metabolic exchange between the cytosol and the IMS (219, 220). MOMP disrupts the overall mitochondrial homeostasis, and prevents the proper management of Ca^2+^, leading to an uncontrolled accumulation of cytosolic Ca^2+^ into mitochondrial matrix and subsequent sustained PTP opening (221).

The sustained opening of PTP allows the diffusion of solutes up to 1.5 kDa across the IMM, which causes matrix swelling and rupture of the OMM, leading to subsequent MOMP (Fig. 2) (210). When the OMM remains intact during mitochondrial stress, matrix swelling is constrained by the outer membrane’s structure, and MPT can occur in a transient or partial manner (222). The PTP opening is readily and fully reversible in intact mitochondria (223) and human cells (222). Its transient or flickering open state is an intrinsic property of mitochondria and a spontaneous event that undergoes in single mitochondria of living cells (224).

Opening of the PTP was found to be refractory to inducers in mitoplasts; the OMM may play a role in regulating this process, but remains to be further investigated (159). VDAC has been suggested to be a putative component of PTP, but this hypothesis has been ruled out based on genetic inactivation studies (225, 226). BAX/BAK has been implicated in the regulation of the PTP because BAX/BAK-null mitochondria are resistant to PTP opening (227, 228, 229). Even though they are essential for PTP opening-induced MOMP, they are not directly responsible for the regulation or formation of the PTP (14).

MTCH2 functions as a crucial facilitator of tBID recruitment to the mitochondria and ultimately induces MOMP to trigger apoptosis (Fig. 2) (151, 152, 153, 154, 155). MTCH2 acts as a repressor of OXPHOS and mitochondrial metabolism (230, 231, 232). MTCH2 is shown to be a direct regulator of mitochondrial fusion, and loss of MTCH2 causes mitochondrial fragmentation (154, 233, 234). MTCH2’s multifaceted roles in mitochondrial homeostasis, dynamics, and metabolism may indirectly lower the threshold for the PTP activation by driving metabolic dysfunction, mtROS production, and Ca^2+^ overload (235). The very recent studies show that MTCH2 binds to CyPD and promotes the dimerization of F-ATP synthase *via* interaction with subunit j, thus regulating the PTP activity (Fig. 2) (17). This work demonstrates that the interplay between MTCH2 and subunit j of F-ATP synthase coordinates MOMP and the PTP opening to mediate the occurrence of MPT (17). This finding suggests that MPT can be coordinately mediated by the interaction(s) between the IMM and OMM proteins, and MOMP induced by the PTP opening is not just a kind of mechanical rupture of the OMM (Fig. 2) (17). This discovery provides an explanation why the OMM is required for opening of the PTP and a molecular mechanism through which OMM protein(s) regulate the PTP.

### MPT in redox homeostasis and ferroptosis

MPT mediated by the PTP acts as a highly regulated and ROS-sensitive molecular switch that balances mitochondrial redox homeostasis (15, 210). The PTP is highly sensitive to matrix Ca^2+^, ROS, redox state of protein thiols, ATP/ADP and NADH/NAD^+^ balance (210, 236). The PTP is a nonselective channel that provides a release pathway for Ca^2+^ and ROS as well as mitochondrial metabolites (18). The PTP regulates redox homeostasis and cell death depending on its open state (Fig. 2) (16).

As described earlier, the tPTP acts as a protective regulator of the redox state through its transient or flickering openings of the pore under conditions of mild or physiologically induced mitochondrial stress (Fig. 1) (16, 17). The tPTP enables the efflux of excess Ca^2+^ from the matrix to avoid Ca^2+^ overload, a major driver of oxidative stress (237). The tPTP contributes to the dissipation of Δψm, accelerates the flow of electrons and prevents over-reduction of the ETC, reducing mtROS production (158, 210). By relieving excessive Δψm and over-reduced ETC, the tPTP suppresses RET and electron leakage, thereby minimizing mtROS generation (158, 210). By relaxing the pressure on ETC, the tPTP also accelerates NADH oxidation and restores NAD^+^ availability, maintaining NADH/NAD^+^ balance and an optimal redox environment, thereby sustaining mitochondrial metabolism (238, 239). The tPTP mitigates the buildup of mtROS that preserves GSH levels and maintains cytosolic GSH/GSSG balance (15, 36). The tPTP modulates the frequency and amplitude of ROS pulses, known as mitoflashes, to orchestrate redox-sensitive signaling pathways involved in cell differentiation, adaptation, and survival (15, 224). The RIRR mediated by tPTP constitutes an adaptive housekeeping function by the timely release of mtROS, preventing excessive mtROS accumulation, allowing mitochondria to signal to the rest of the cell to balance redox states and maintain metabolic stability (15, 16). The tPTP may also regulate intracellular redox homeostasis by mediating mitochondria-organelle interactions (18, 86).

The sustained opening of PTP leads to collapse of Δψm, depletion of ATP, the burst of ROS, and mitochondrial swelling followed by release of pro-apoptotic proteins (14, 16, 18). The PTP opening-induced mitochondrial dysfunction amplifies mtROS production and forces a shift from physiological signaling to pathological redox collapse (15, 210). The collapse of Δψm and mitochondrial dysfunction prevents the conversion of NADH to NADPH by NADP^+^-dependent enzymes NNT, isocitrate dehydrogenase 2 and malic enzyme 3 (240, 241). NADPH depletion halts the regeneration of GSH and reduces the activity of Trx reductase, which directly causes a collapse in the cellular primary antioxidant systems, leading to severe oxidative stress (242, 243). Under certain conditions, the GSH and Grx systems can act as a backup for the Trx systems, but if both NADPH and GSH are severely depleted, this redundancy fails, leading to rapid cell death (244, 245). The increased consumption of NADPH and rapid oxidation of GSH to GSSG by massive mtROS production cause redox imbalance. Mitochondrial dysfunction and the PTP-dependent release of mtROS into the cytosol activate NOXs, driving a positive feedforward cycle between NOXs and mtROS that disrupts redox homeostasis (246, 247). Thus, the sustained opening of PTP converts mitochondria from NADPH-generating, redox-protective organelles into ROS-amplifying, antioxidant-depleted systems (Fig. 2).

As a release channel, the tPTP contributes to ROS homeostasis as well as chemoresistance (16). MTCH2 maintains mitochondrial architecture and represses OXPHOS, which reduces ROS generation and inhibits ferroptosis (130, 231). Silencing or knockdown of MTCH2 increases the sensitivity of cells to ferroptosis-inducing agents and induces ferroptosis (248, 249, 250). The most recent work suggests that CyPD favors the interaction between MTCH2 and subunit j of F-ATP synthase, which promotes dimerization of the enzyme and modulates the PTP activity (17). We further propose that MTCH2 cooperates with F-ATP synthase, coordinating MOMP and the PTP opening to mediate the occurrence of MPT (17). RSL3 drives ferroptosis by inactivating GPx4 and increasing intracellular ROS levels (92). Knockdown of CyPD, MTCH2 or subunit j impairs the tPTP and markedly promotes RSL3-induced ferroptosis, which could be prevented by mitochondrial superoxide scavenger MitoTEMPO (17). The tPTP allows the release of mtROS into cytosol, which prevents accumulation of mtROS within the matrix and mitochondrial lipid peroxidation; thus, the tPTP acts as another mitochondrial defense system against ferroptosis (17). Under physiological conditions, the tPTP reduces mtROS production and maintains NADPH-dependent antioxidant systems, which can delay or suppress ferroptosis (29, 251). Conversely, the sustained opening of PTP leads to NADPH collapse, GSH depletion, GPX4 dysfunction, and ROS amplification, which accelerates lipid peroxidation and thus ferroptosis (Fig. 2) (29, 251).

## Conclusion

Mitochondria are central hubs in maintaining redox homeostasis and directing cell death. Mitochondria are the primary intracellular source of ROS, and the efflux of mtROS can activate cytoplasmic NOXs that increase cellular ROS production. Mitochondria develop adaptive antioxidant systems to counteract the extra ROS, acting as a redox sink for both internal and exogenous ROS (Fig. 1). Mitochondria play critical roles in diverse death pathways such as apoptosis, necrosis and ferroptosis.

The PTP is a critical molecular switch that balances mitochondrial redox homeostasis. Depending on the open states of PTP, it can convert from a defender against mitochondrial redox stress and ferroptosis into a ROS amplifier and cell death promoter (Fig. 2). The tPTP mitigates the buildup of mtROS and provides a reversible efflux pathway for mtROS, preventing excessive mtROS accumulation in the matrix. Thus, the tPTP functions as a mitochondrial defense system against oxidative stress and ferroptosis, favoring redox homeostasis and cell survival. Conversely, the sustained opening of PTP induces mitochondrial dysfunction, amplifies mtROS production and depletes antioxidant systems, leading to severe oxidative stress. MOMP causes the uncoupling of OXPHOS, leading to increased mtROS production and oxidative stress (Fig. 1). The PTP opening and MOMP interact in a bidirectional manner, and coordinately mediate the MPT. MOMP allows the release of proapoptotic factors like cytochrome *c* to enter the cytosol, leading to cell death. MOMP can occur without significant matrix swelling, while the sustained opening of PTP causes matrix swelling and rupture of the OMM, leading to subsequent MOMP. When matrix swelling is constrained by an intact OMM, the PTP opens in a reversible and flickering mode referred to as tPTP, and MPT occurs in a transient or partial manner. Thus, limiting MOMP can regulate the open states of the PTP.

Emerging studies suggest that the PTP forms from F-ATP synthase. MTCH2 is a crucial outer membrane receptor of tBID and facilitates MOMP. The interplay between MTCH2 and subunit j of F-ATP synthase coordinates MOMP and the PTP opening to mediate the occurrence of MPT (Fig. 2). Uncovering the regulatory mechanisms of MPT provides promising therapeutic strategies for multiple pathologies such as cancer and neurodegenerative diseases.

## Conflicts of interest

The authors declare that they have no conflicts of interest with the contents of this article.
